# Heterogeneity in response to GLP-1 receptor agonists in type 2 diabetes in real-world clinical practice: insights from the DPV register – an IMI-SOPHIA study

**DOI:** 10.1007/s00125-025-06448-w

**Published:** 2025-05-22

**Authors:** Martin Heni, Lisa Frühwald, Wolfram Karges, Michael Naudorf, Kathrin Niemöller, Frank Pagnia, Jörg Reindel, Jochen Seufert, Gisa Ufer, Christian Wagner, Reinhard W. Holl, Nicole Prinz

**Affiliations:** 1 Division of Endocrinology and Diabetology, Department of Internal Medicine I, Ulm University Hospital, Ulm, Germany; 2 Institute for Clinical Chemistry and Pathobiochemistry, University Hospital Tübingen, Tübingen, Germany; 3 5th Medical Department, Endocrinology, Rheumatology and Acute Geriatrics, Hospital Ottakring, Vienna, Austria; 4 Division of Endocrinology and Diabetes, Medical Faculty, RWTH Aachen University, Aachen, Germany; 5 Diabetes Center Lindlar, Lindlar, Germany; 6 Knappschaftskrankenhaus Dortmund, Department of Internal Medicine V, Dortmund, Germany; 7 Specialised Diabetes Practice, Memmingen, Germany; 8 Department of Diabetology, Metabolic Disorders and Nephrology, Hospital Karlsburg, Karlsburg, Germany; 9 Division of Endocrinology and Diabetology, Department of Medicine II, Medical Center – University of Freiburg, Faculty of Medicine, University of Freiburg, Freiburg, Germany; 10 Specialised Diabetes Practice, Saaldorf-Surheim, Germany; 11 Institute of Epidemiology and Medical Biometry, CAQM, University of Ulm, Ulm, Germany; 12 German Centre for Diabetes Research (DZD), Munich-Neuherberg, Germany

**Keywords:** Body weight, GLP-1 receptor agonists, HbA_1c_, Heterogeneity, Real life

## Abstract

**Aims/hypothesis:**

Glucagon-like peptide-1 receptor agonists (GLP-1 RAs) are a cornerstone in type 2 diabetes management. In this study we evaluated heterogeneity in body weight and glycaemic responses to the initiation of liraglutide, semaglutide or dulaglutide in real-world clinical practice.

**Methods:**

Data from 4467 adults with type 2 diabetes in the Diabetes Patient Follow-up (DPV) registry were analysed, focusing on changes in HbA_1c_ and body weight over 6 months following initiation of a GLP-1 RA. We categorised participants based on their response: HbA_1c_ reduction only, weight loss only, both or neither. This analysis was part of the IMI-Stratification of Obesity Phenotypes to Optimize Future Obesity Therapy (IMI-SOPHIA) project.

**Results:**

At 6 months’ follow-up the median absolute HbA_1c_ reduction was 5.3 mmol/mol (IQR 13.9, −1.0) (0.49% [1.27, −0.09]) and relative body weight reduction was 1.43% (4.26, 0). Only 14% of participants achieved meaningful reductions in both HbA_1c_ (absolute reduction ≥5.5 mmol/mol [0.5%]) and body weight (relative reduction ≥5%). Men and those with a higher baseline HbA_1c_ were more likely to show an HbA_1c_ only response (36% of participants; both *p*<0.001), while older individuals and those with a longer diabetes duration were more likely to experience a weight-only response (7% of participants; both *p*<0.001). Higher baseline body weight and lower eGFR (both *p*<0.05) correlated with greater weight reduction, whereas lower baseline HbA_1c_ and longer diabetes duration were linked to smaller HbA_1c_ reductions (both *p*<0.001).

**Conclusions/interpretation:**

There is significant heterogeneity in responses to GLP-1 RA therapy among individuals with type 2 diabetes in routine clinical practice. However, in our study a substantial proportion achieved a reduction in either body weight or HbA_1c_. Future studies should explore why some individuals achieve either weight loss or HbA_1c_ reduction but not both.

**Graphical Abstract:**

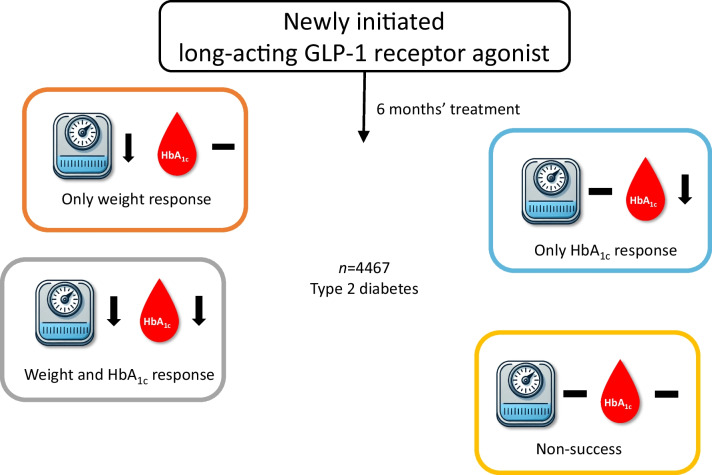

**Supplementary Information:**

The online version of this article (10.1007/s00125-025-06448-w) contains peer-reviewed but unedited supplementary material.


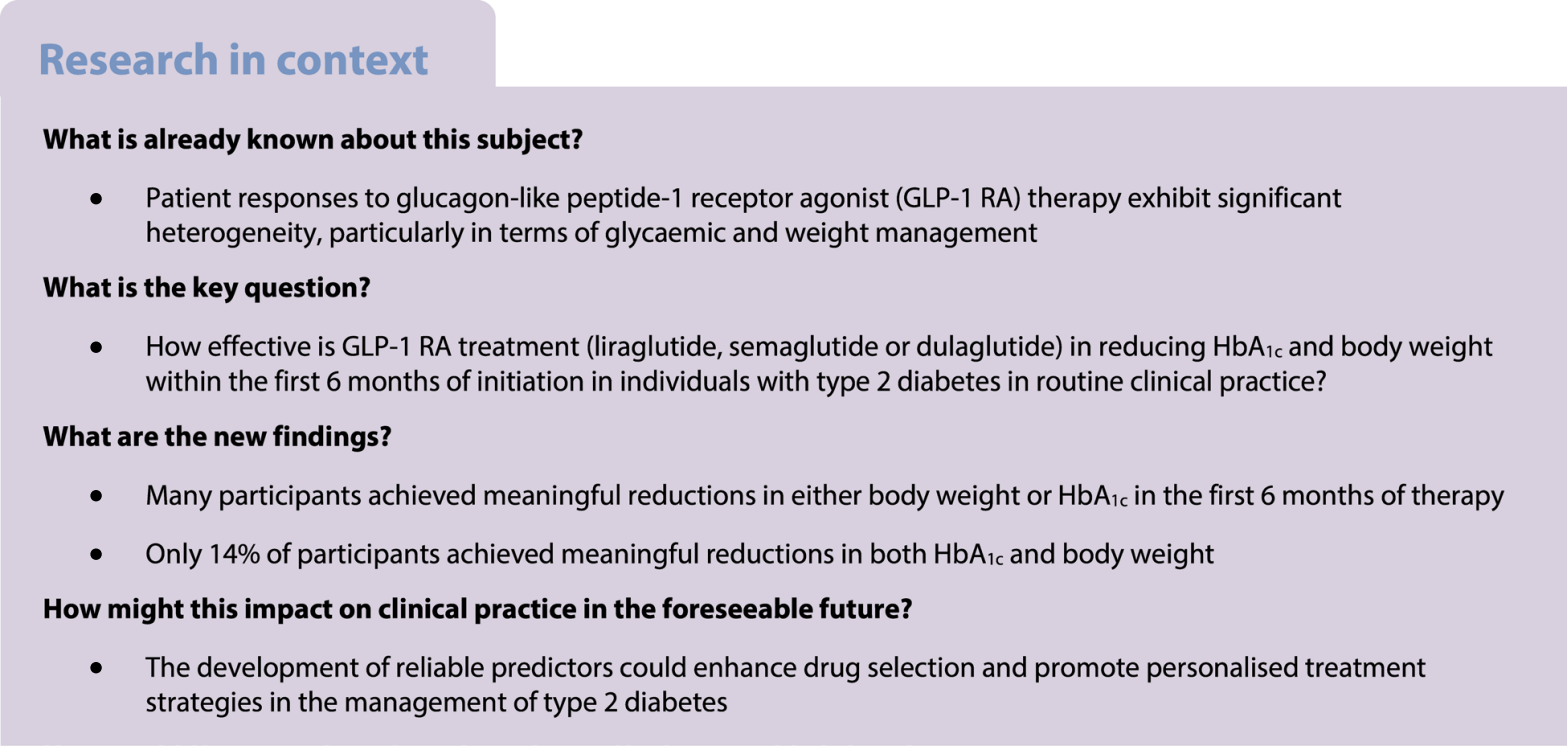


## Introduction

Glucagon-like peptide-1 receptor agonists (GLP-1 RAs) are a cornerstone in type 2 diabetes management, improving glucose-stimulated insulin secretion, slowing gastric emptying and reducing blood glucose levels [1, 2]. Besides these well-established effects, they promote weight loss, mainly through their action in the brain [1, 2], and are therefore also approved for weight management, even in those with normal glucose regulation. In addition to reducing HbA_1c_ and body weight, accumulating evidence underscores their role in reducing risk for diabetes complications and even mortality [2]. GLP-1 RAs show promise in other conditions linked to diabetes, including hypertension, hyperlipidaemia, liver steatosis and subclinical inflammation [2].

However, despite their efficacy, individual responses to GLP-1 RAs vary significantly, particularly in terms of glycaemic and weight management [3–6], and this variability in real-world clinical practice, as well as clinical features linked to this, still represent a critical gap in our knowledge. Gaining insights into the heterogeneity could contribute to precision medicine strategies in diabetes care. Currently, precision medicine in diabetes emphasises patient-specific phenotypic data and distinct diabetes endotypes [7]. Incorporating treatment response variability, such as responses to GLP-1 RAs, could further refine and enhance these personalised treatment approaches. In this study, we analysed HbA_1c_ and body weight changes in the large Diabetes Patient Follow-up (DPV) dataset, focusing on the first 6 months after initiation of a long-acting GLP-1 RA. Success was clinically defined as a 5% relative body weight reduction and a 5.5 mmol/mol (approx. 0.5%) absolute HbA_1c_ reduction. The analysis was part of the IMI-Stratification of Obesity Phenotypes to Optimize Future Obesity Therapy (IMI-SOPHIA) project.

## Methods

The DPV registry is a long-term, prospective registry collecting real-world data on diabetes diagnoses, management and outcomes across four European countries (Germany, Austria, Switzerland and Luxembourg) [8, 9]. It aggregates anonymised data from currently 521 centres on nearly 700,000 individuals with diabetes, allowing multicentre analyses and benchmarking of diabetes care. Data are pseudonymised and transferred biannually to Ulm University, Germany, where incomplete or implausible data are verified with the respective centres [8, 9]. The DPV registry is representative of pediatric diabetes care and adults with diabetes treated in diabetes-specialised practices in the four participating European countries [10]. Race and ethnicity are not well documented in the DPV registry for the participant group included and were therefore not analysed in this study. The DPV initiative and pseudonymised data analysis are approved by the ethics committee of Ulm University, Germany (314/21), and by local ethics boards of participating centres.

### Study population

**Fig. 1:**
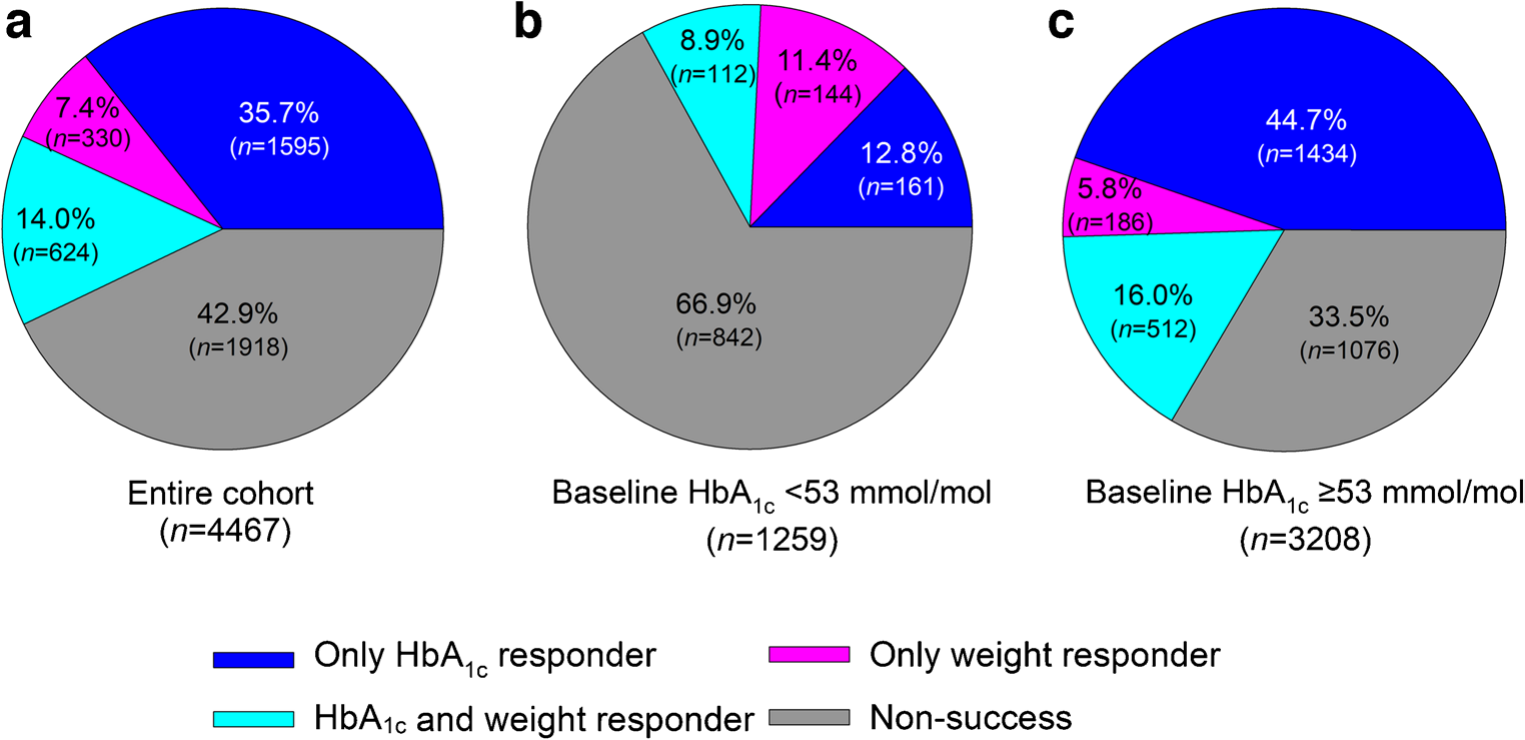
Heterogeneous responses to GLP-1RAs in the entire cohort (**a**) and stratified by baseline HbA_1c_ (<53 mmol/mol [**b**]; ≥53 mmol/mol [**c**])

In the current analysis, participants had to be diagnosed with type 2 diabetes and be aged ≥18 years when starting a GLP-1 RA (a flow-chart of the selection of the study cohort is provided in electronic supplementary material [ESM] Fig. 1). We focused on long-acting GLP-1 RAs and therefore excluded individuals receiving lixisenatide (*n*=69) and exenatide (*n*=667). The final cohort included 4467 individuals, categorised into four subgroups depending on their change in weight and HbA_1c_ within the first 6 months after GLP-1 RA initiation: individuals reducing one response variable only (either absolute HbA_1c_ reduction ≥5.5 mmol/mol [≥0.5%] or relative body weight reduction ≥5%), individuals reducing both variables or individuals reducing neither variable.

The distribution of GLP-1 RAs among participants is shown in ESM Fig. 2. Sex was self-reported.

### Variables

Glycaemic control was assessed using HbA_1c_, standardised to the DCCT reference [11]. Hypertension and dyslipidaemia were defined as described previously [8, 9]. History of stroke, myocardial infarction, heart failure, ischaemic heart disease, angina pectoris, peripheral artery disease, diabetic foot syndrome and neuropathy was assessed via ICD-10 diagnostic codes (https://icd.who.int/browse10/2019/en), foot examinations or neuropathy screens. Nephropathy was defined as a history of eGFR <60 ml/min per 1.73 m^2^ (calculated using the Chronic Kidney Disease Epidemiology Collaboration [CKD-EPI] formula [12]), micro- or macroalbuminuria, kidney transplant or dialysis. Microalbuminuria and retinopathy were identified through routine screening [8]. Macrovascular complications consisted of myocardial infarction, stroke, heart failure, angina pectoris, peripheral artery disease, ischaemic heart disease and diabetic foot syndrome. Microvascular complications encompassed microalbuminuria, retinopathy and neuropathy. Smoking was self-reported and documented if recorded at least once.

### Statistical analysis

Analyses were performed using SAS (version 9.4, TS1M7, SAS Institute). Participant characteristics were compared between subgroups using Kruskal–Wallis and χ^2^ tests, with results presented as medians with IQRs or as proportions and absolute numbers.

Multinomial logistic regression was used to assess subgroup membership likelihood based on baseline parameters, with separate models for sex, age, diabetes duration, BMI, HbA_1c_ and eGFR. A basic model adjusted for sex, age and diabetes duration at GLP-1RA initiation was further refined by adding BMI, HbA_1c_, additional glucose-lowering medication, vascular complications, hypertension, dyslipidaemia and smoking status, each separately. The subgroup with both HbA_1c_ and weight reduction was used as a reference. Additionally, multinomial logistic analyses were repeated comparing the only weight reduction vs only-HbA_1c_ reduction response groups. Results are presented as ORs with 95% CIs.

Linear regression analyses were used to evaluate the impact of baseline characteristics on weight or HbA_1c_ change, with results presented as standardised β-coefficients with 95% CIs. For continuous parameters, the coefficient was interpreted as the number of SDs of change in the outcome variable for 1 SD change in the explanatory variable, holding the other variables constant.

A two-tailed *p* value <0.05 was considered significant. Bonferroni–Holm correction was applied for multiple comparisons.

## Results

The study cohort had a median age at GLP-1RA initiation of 60.0 years (IQR 52.1, 68.1), with a median BMI of 34.9 kg/m^2^ (31.0, 40.0) and a median HbA_1c_ of 60 mmol/mol (52, 72) (7.7% [6.9, 8.7]). Full baseline characteristics of the study cohort are provided in ESM Table 1. In total, 1890 of 4467 participants changed co-medication within the follow-up period. Aside from a decrease in additional dipeptidyl-peptidase 4 inhibitor (DPP-4i) use from 31.5% to 14.3% of participants, rates for other co-medications were relatively stable, with <5% of participants changing other co-medications (insulin, +1%; metformin, −0.1%; sodium–glucose cotransporter 2 inhibitors [SGLT-2is], +1.5%; sulfonylureas/glinides, −1.8%; thiazolidines, −0.4%).

**Fig. 2:**
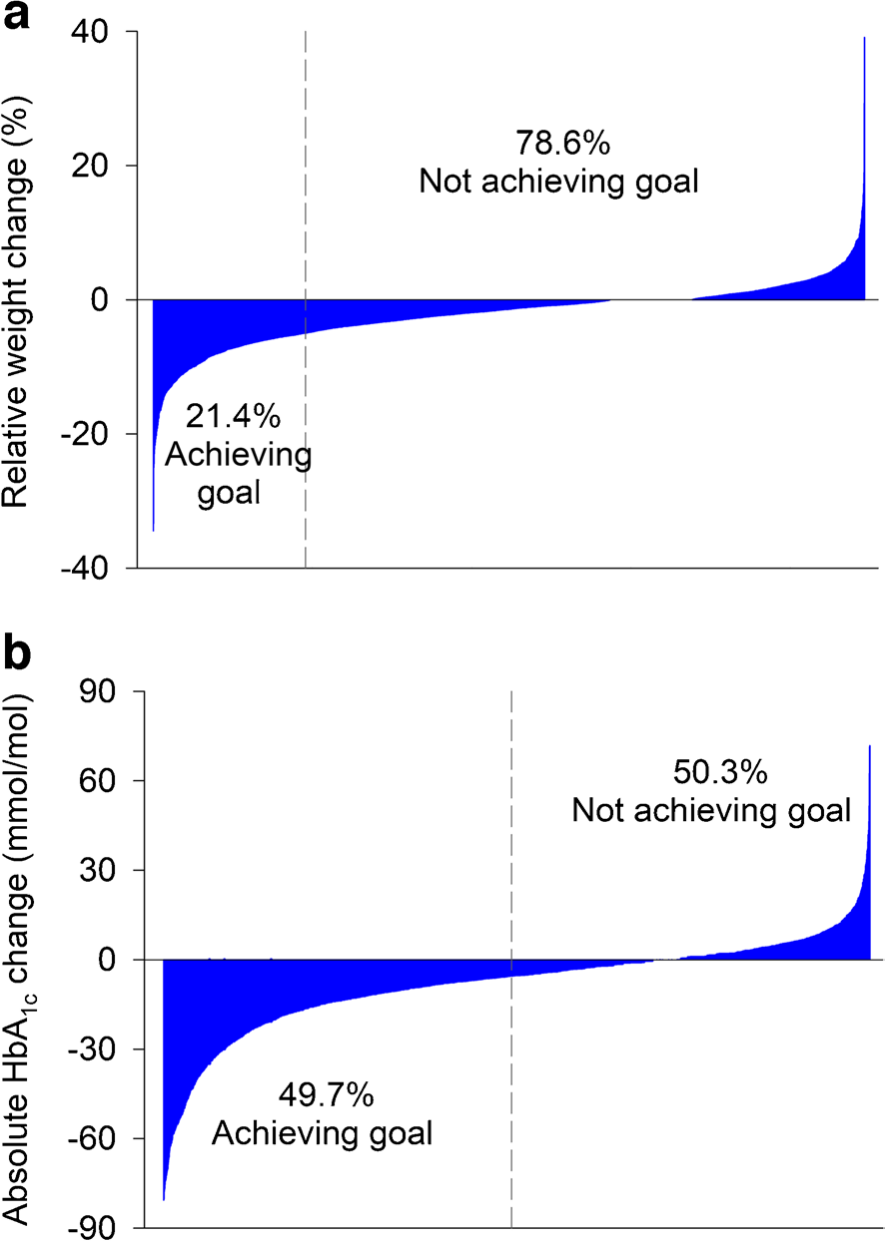
Waterfall plots for individuals’ relative change in body weight (**a**) and absolute change in HbA_1c_ (**b**) up to 6 months after initiation of liraglutide, semaglutide or dulaglutide (*n*=4467). The grey dashed lines represent cut-offs for the proportions of individuals achieving a successful reduction in either body weight (relative reduction ≥5%) or HbA_1c_ (absolute reduction ≥5.5 mmol/mol or 0.5%)

During the 6 month follow-up, participants achieved a median absolute HbA_1c_ reduction of 5.3 mmol/mol (IQR 13.9, −1.0) (0.49% [1.27, −0.09]) and a median relative weight reduction of 1.43% (4.26, 0.00). In total, 14% of participants achieved both HbA_1c_ and weight reduction (‘HbA_1c_ and weight responder’). Another 35.7% achieved HbA_1c_ reduction only, with no meaningful weight reduction (‘only HbA_1c_ responder’), while 7.4% achieved weight reduction only, with no meaningful HbA_1c_ response (‘only weight responder’). The remaining 42.9% showed no significant reductions in either measure (‘non-success’). The heterogeneous response stratified by median baseline HbA_1c_ (<53 mmol/mol vs ≥53 mmol/mol) is illustrated in Fig. 1. Baseline characteristics of the four subgroups are provided in ESM Table 1. Figure 2 presents waterfall plots for individuals’ relative change in body weight and absolute change in HbA_1c_ (ESM Fig. 3 provides corresponding data for individuals using lixisenatide and exenatide).

**Fig. 3:**
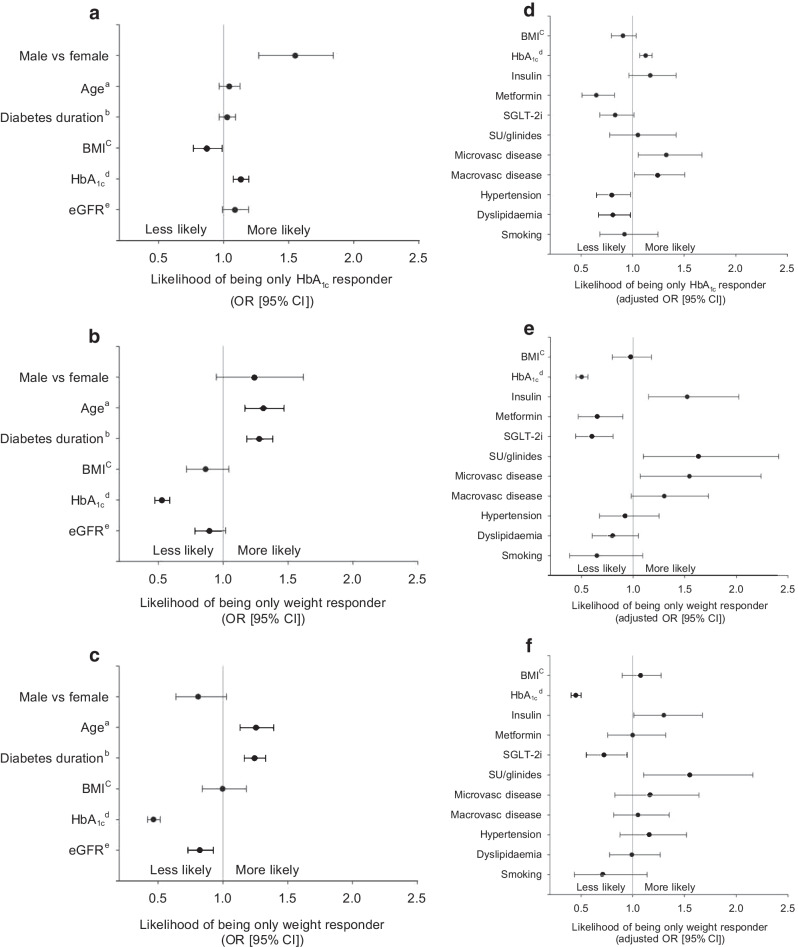
Likelihood of group membership. (**a**, **d**) Likelihood of belonging to the only HbA_1c_ responder group vs the HbA_1c_ and weight responder group, (**b**, **e**) likelihood of belonging to the only weight responder group vs the HbA_1c_ and weight responder group and (**c**, **f**) likelihood of belonging to the only weight responder group vs the only HbA_1c_ responder group. (**a**–**c**) show results from univariate logistic regression analyses and (**d**–**f**) show results from separate multivariate logistic regression models, each adjusted for sex, age and diabetes duration at the time of GLP-1 RA initiation. ^a^per 10 year increase; ^b^per 5 year increase; ^c^per 10 kg/m^2^ increase; ^d^per 10 mmol/mol increase; ^e^per 0.1 mg/min per 1.73m^2^ increase (all other variables are yes vs no). Macrovasc, macrovascular; microvasc, microvascular; SU, sulfonylurea

Factors associated with belonging to the only HbA_1c_ responder group or only weight responder group compared with the HbA_1c_ and body weight were analysed (Fig. 3). Men had a higher likelihood of being in the only HbA_1c_ responder group (OR [95% CI] 1.53 [1.27, 1.84]), as did those with an elevated baseline HbA_1c_ (1.13 [1.07, 1.19], both *p*<0.001). Higher BMI reduced the likelihood of being in the only HbA_1c_ responder group (0.87 [0.76, 0.99], *p*=0.03), but age and diabetes duration showed no associations compared with the group with meaningful responses in both HbA_1c_ and body weight (Fig. 3a, both *p*>0.05). Of the complications and comorbidities investigated, all were significantly linked to membership of the only HbA_1c_ responder group (Fig. 3d).

The likelihood of belonging to the only weight responder group compared with the HbA_1c_ and weight responder group was similar between sexes (*p*=0.12) but increased with age (1.31 [1.17, 1.47], *p*<0.001) and longer diabetes duration (1.28 [1.18, 1.38], *p*<0.001). Higher baseline HbA_1c_ reduced the likelihood of belonging to this group (0.52 [0.47, 0.59], *p*<0.001) (Fig. 3b). The presence of microvascular complications was linked to a higher likelihood of membership of the only weight responder group (Fig. 3e, *p*=0.02).

When comparing the only HbA_1c_ responder and only weight responder groups, older people and those with a longer diabetes duration were more likely to belong to the only weight responder group (1.31 [1.17, 1.47] and 1.24 [1.16, 1.33], respectively; both *p*<0.001). Conversely, higher baseline HbA_1c_ and eGFR were linked to membership in the only HbA_1c_ responder group (2.15 [1.94, 2.39], *p*<0.001, and 1.32 [1.16, 1.51], *p*=0.001, respectively) (Fig. 3c). Complications and comorbidities had no impact on group membership (Fig. 3f).

We next analysed the associations between baseline characteristics and overall weight or HbA_1c_ changes across all groups using linear regression. Standardised β-coefficients (with 95% CIs) are presented in ESM Fig. 4. Weight reduction was most strongly associated with sex, with female participants showing greater reductions. Additionally, higher baseline body weight and lower eGFR were associated with greater weight loss (all *p*<0.05). Higher baseline HbA_1c_ was associated with greater HbA_1c_ reductions, while longer diabetes duration was associated with smaller HbA_1c_ reductions (both *p*<0.001).

## Discussion

We detected marked heterogeneity in glycaemic and weight responses on GLP-1 RA initiation in adult participants with type 2 diabetes under real-world conditions. As the long-acting (more potent) GLP-1 RAs liraglutide, semaglutide and dulaglutide [1, 2] are becoming the standard in treatment, we focused on these agents. A surprising finding was that only 14% of participants achieved marked reductions in both HbA_1c_ and body weight, with many responding primarily in one area only. Factors such as sex, age, HbA_1c_, BMI and diabetes duration at treatment initiation were linked to the likelihood of response.

It is possible that participants in the group with neither weight loss nor glucose reduction discontinued or only sporadically used the drug, so we focused on those who achieved a reduction in either outcome, as it is more likely that these individuals used the drug as prescribed. Among those achieving reductions, the proportion showing meaningful responses in both areas was lower than in clinical trials, which often involve highly selected, motivated populations that might therefore not be representative of the wide spectrum of individuals with type 2 diabetes in routine clinical practice [3]. In addition, the stricter management in trials may explain some of the discrepancy with findings from real-world settings, a common observation in studies of different drug classes [13]. Our data provide some insights into factors influencing treatment response, but developing robust biomarkers may be necessary for precision medicine approaches at the individual level. Although higher baseline HbA_1c_ predictably associates with greater glycaemic reductions, the marked heterogeneity in response (as visualised in Fig. 1) highlights that baseline HbA_1c_ alone does not fully capture treatment variability. This underscores the importance of identifying additional clinical or biological factors that can better predict glycaemic response to GLP-1 RA treatment.

Consistent with most previous trials, participants with higher pretreatment weight were more likely to lose more weight on GLP-1 RA initiation, with women achieving greater weight reductions than men [14–16]. However, despite assumptions that women experience more side effects from GLP-1 RAs than men [15], they were not over-represented in the non-success group in our analysis. Unexpectedly, we found an association between reduced kidney function at baseline and greater weight reduction, which warrants further investigation.

Individuals with diabetes generally experience less weight reduction with GLP-1 RAs than those without diabetes [2, 6]. We found that higher HbA_1c_ was associated with a lower likelihood of marked weight reduction, consistent with a recent meta-analysis suggesting that hyperglycaemia may impact weight management [17]. Further research is needed to explore how high glucose levels might reduce the weight loss response to GLP-1 RAs.

Our findings confirm that longer diabetes duration is associated with a lower glycaemic response to GLP-1 RAs [3, 6, 16], probably due to declining beta cell function, as intact insulin secretion is necessary for a robust glycaemic response to this drug class [1, 3]. Genetic factors may also influence the glycaemic response to GLP-1 RAs, although their impact is uncertain, while a genome-wide study did not find a link between genetic variants and weight loss response to this drug class [3], suggesting that different mechanisms are involved in the glycaemic and weight loss effects of GLP-1 RAs.

One strength of our analysis is its focus on individuals newly initiated on GLP-1 RAs, minimising prescription bias. However, the observational nature of our study limits the availability of variables such as treatment adherence or lifestyle factors, which may contribute to response heterogeneity. In future studies, it will be interesting to explore the heterogeneity in response to new co-agonist drugs, which may have even greater glycaemic and weight loss effects [2].

In conclusion, our analysis reveals substantial variability in GLP-1 RA responses in individuals with type 2 diabetes in routine clinical practice, with only a minority of individuals achieving both glycaemic and weight reductions in the first 6 months of treatment. The real-world disparities compared with clinical trials underscore the need for broader studies to better predict response and guide individualised treatment strategies in type 2 diabetes.

## Supplementary Information

Below is the link to the electronic supplementary material.

ESM (PDF 524 KB)

## Data Availability

The data are not publicly available because they contain information that could compromise research participant privacy/consent.

## Abbreviations

DPV: Diabetes Patient Follow-up
GLP-1 RA: Glucagon-like peptide-1 receptor agonist
SGLT-2i: Sodium–glucose cotransporter 2 inhibitor
