# Supplementary material for: Heterogeneity in response to GLP-1 receptor agonists in type 2 diabetes in real-world clinical practice: insights from the DPV register – an IMI-SOPHIA study
Source: Diabetologia. 2025 May 22;68(8):1666–73. doi: 10.1007/s00125-025-06448-w (PMC12245949; doi:10.1007/s00125-025-06448-w)

Electronic supplementary material to Heni et al.

"Heterogeneity in response to GLP-1 receptor agonists in type 2 diabetes in real-world clinical practice: insights from the DPV register – an IMI-SOPHIA study"

ESM Table 1 Baseline characteristics of individuals using liraglutide, semaglutide, or dulaglutide, and separated by subgroups of response.

	**All**	**HbA_{1c} and weight responder** (reference)	**Only HbA_{1c}-responder**	*P value*[3]	**Only weight-responder**	*P value*[4]	*P-value*[5]	**Non-success**	*P-value*[6]
N, %	4467 (100)	624 (14.0)	1595 (35.7)		330 (7.4)			1918 (42.9)	
Age, years	60.0 (52.1–68.1)	58.1 (50.4–66.5)	58.6 (50.9–67.1)	1.00	62.7 (54.4–70.3)	**<0.001**	**<0.001**	61.5 (53.3–68.9)	**<0.001**
Diabetes duration, years	8.9 (3.9–15.0)	7.7 (2.7–13.9)	7.8 (3.1–13.8)	1.00	11.8 (6.1–17.4)	**<0.001**	**<0.001**	9.8 (4.6–15.9)	**<0.001**
Females, %	45.4	53.5	43.0	**<0.001**	48.2	1.00	1.00	44.3	**0.001**
BMI, kg/m²	34.9 (31.0–40.0)	35.8 (31.6–40.7)	34.9 (31.1–40.1)	0.787	35.3 (30.9–39.9)	1.00	1.00	34.7 (30.8–39.8)	**0.019**
HbA_{1c}, mmol/mol	60 (52–72)	65 (56–77)	68 (60–81)		54 (47–63)			55 (48–63)	
HbA_{1c}, %	7.7 (6.9–8.7)	8.1 (7.3–9.2)	8.4 (7.6–9.6)	**<0.001**	7.1 (6.4–7.9)	**<0.001**	**<0.001**	7.2 (6.6–7.9)	**<0.001**
Insulin dose, U/kg*d	0.4 (0.2–0.7)<br>n=1,926	0.4 (0.2–0.6)<br>n=242	0.4 (0.2–0.7)<br>n=675	0.787	0.5 (0.3–0.8)<br>n=173	**<0.001**	**0.003**	0.4 (0.3–0.7)<br>n=836	**0.006**
Insulin, N (%)	2,171 (48.6)	270 (43.3)	764 (47.9)	1.00	195 (59.1)	**<0.001**	**0.006**	940 (49.0)	0.227
Metformin, N (%)	3,390 (75.9)	519 (83.2)	1,219 (76.4)	**0.016**	246 (74.6)	**0.038**	1.00	1,408 (73.4)	**<0.001**
DPP-4i, N (%)	1,407 (31.5)	207 (29.3)	493 (30.9)	1.00	116 (35.2)	0.911	1.00	616 (32.1)	1.00
SGLT-2i, N (%)	1,300 (29.1)	213 (34.1)	498 (31.2)	1.00	84 (25.5)	0.135	0.839	506 (26.4)	**0.005**
SU/Glinides, N (%)	554 (12.4)	65 (10.4)	175 (11.0)	1.00	53 (16.1)	0.247	0.249	261 (13.6)	0.538
Thiazolidinediones, N (%)	156 (3.5)	11 (1.8)	49 (3.1)	1.00	27 (8.2)	**<0.001**	**<0.001**	71 (3.7)	0.316
Total cholesterol, mmol/l	4.8 (4.0–5.6)<br>n=3179	4.8 (4.0–5.7)<br>n=443	4.9 (4.1–5.6)<br>n=1160	1.00	4.7 (3.9–5.6)<br>n=230	1.00	1.00	4.7 (4.0–5.5)<br>n=1346	1.00
HDL cholesterol, mmol/l	1.1 (1.0–1.4)<br>n=2772	1.1 (1.0–1.3)<br>n=372	1.1 (0.9–1.3)<br>n=1010	1.00	1.1 (0.9–1.4)<br>n=199	1.00	1.00	1.2 (1.0–1.4)<br>n=1191	1.00
LDL cholesterol, mmol/l	2.8 (2.2–3.5)<br>n=2817	2.9 (2.2–3.6)<br>n=398	2.9 (2.3–3.6)<br>n=993	1.00	2.7 (2.1–3.4)<br>n=217	0.975	0.839	2.8 (2.1–3.5)<br>n=1209	0.743
Dyslipidaemia, N (%)	2,573 (57.6)	379 (60.7)	904 (56.7)	1.00	194 (58.8)	1.00	1.00	1095 (57.1)	0.912
Systolic BP, mmHg	138 (128–149)<br>n=4197	138 (130–150)<br>n=582	140 (130–150)<br>n=1481	1.00	139 (130–147)<br>n=314	1.00	1.00	135 (127–146)<br>n=1820	0.330
Diastolic BP, mmHg	80 (76–90)<br>n=4190	82 (77–90)<br>n=582	82 (77–90)<br>n=1478	1.00	80 (75–90)<br>n=314	0.304	0.818	80 (75–88)<br>n=1816	**0.001**
Hypertension, N (%)	3,096 (69.3)	455 (72.9)	1102 (69.1)	1.00	246 (74.6)	1.00	0.839	1295 (67.5)	0.214
eGFR, mg/min per 1.73 m²	0.70 (0.62–0.79)<br>n=3603	0.69 (0.62–0.80)<br>n=516	0.72(0.63–0.80)<br>n=1290	1.00	0.69 (0.61–0.77)<br>n=273	1.00	**0.034**	0.70 (0.62–0.79)<br>n=1,524	1.00
Microvascular disease[1], N (%)	3,645 (81.6)	472 (75.6)	1292 (81.0)	0.147	283 (85.8)	**0.008**	0.839	1598 (83.3)	**<0.001**
Macrovascular disease[2], N (%)	2417 (54.1)	292 (46.8)	850 (53.3)	0.165	194 (58.8)	**0.012**	1.00	1080 (56.3)	**0.001**
Smoking, %	16.2<br>n=2909	18.8<br>n=409	17.9<br>n=1086	1.00	11.2<br>n=197	0.304	0.521	14.7<br>n=1217	0.538

Presented are median (IQR) or absolute numbers/proportions. BMI body mass index, DPP-4i Dipeptidyl-peptidase 4 inhibitors, eGFR estimated glomerular filtration rate, HbA_{1c} hemoglobin A_{1c}, HDL high density lipoprotein, LDL low density lipoprotein, SGLT-2i Sodium glucose linked transporter 2 inhibitors, SU sulfonylurea, [1]microalbuminuria and/or retinopathy and/or any neuropathy, [2]myocardial infarction and/or stroke and/or heart failure and/or angina pectoris and/or peripheral artery disease and/or ischaemic heart disease and/or diabetic foot syndrome. Groups were compared Kruskal-Wallis or Chi-squared tests, as appropriate. P values are adjusted by Bonferroni-Holm-correction for multiple comparisons. [3]p-value compares only HbA_{1c} vs. HbA_{1c} and weight responder, [4]p-value compares only weight vs. HbA_{1c} and weight responder, [5]p-value compares only weight vs. only HbA_{1c} responder, [6]p-value compares non-success vs. HbA_{1c} and weight responder.

ESM Fig. 1 Selection of study cohort from the prospective diabetes follow-up register, DPV. *a maximum time-period of up to 6 months prior to the first GLP-1RA use was defined as index date; the time-period 3 months up to 9 months after first GLP-1RA use was defined as 6 months follow-up (FU); in case of multiple visits, data have been aggregated as median values.

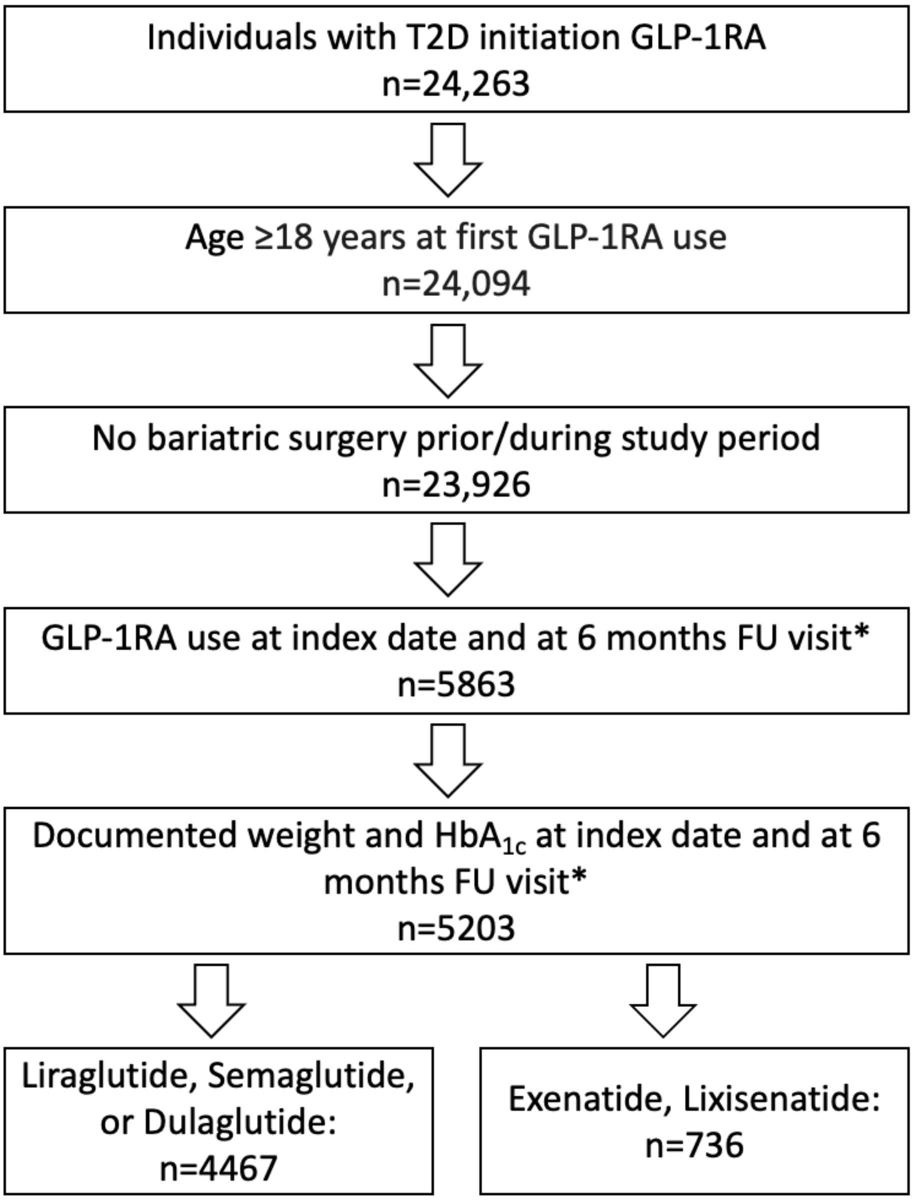

ESM Fig. 2 The distribution of GLP-1 RA among participants.

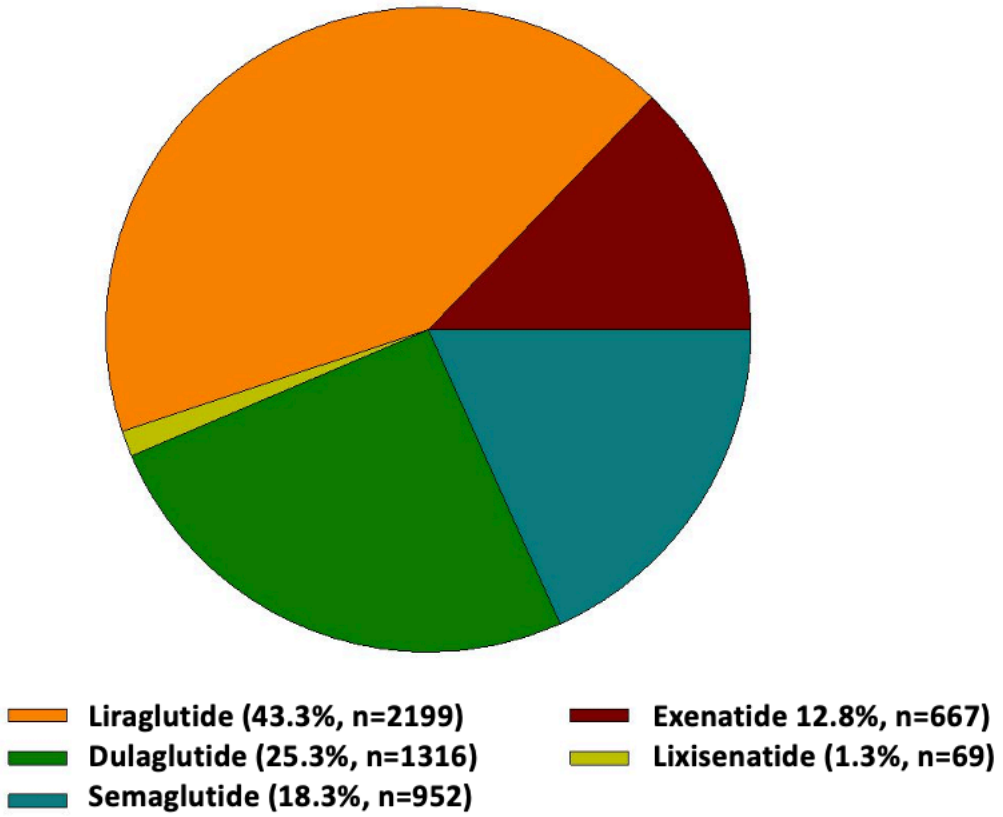

ESM Fig. 3 Waterfall plots for individuals' relative change of body weight (**a**) and absolute change of HbA_{1c} (**b**) up to 6 months after initiation of lixisenatide or exenatide (n=736). Grey dashed line represents cut-off for proportion of individuals achieving successful reduction in either body weight (≥5%) or HbA_{1c} (≥5.5 mmol/mol or 0.5%).

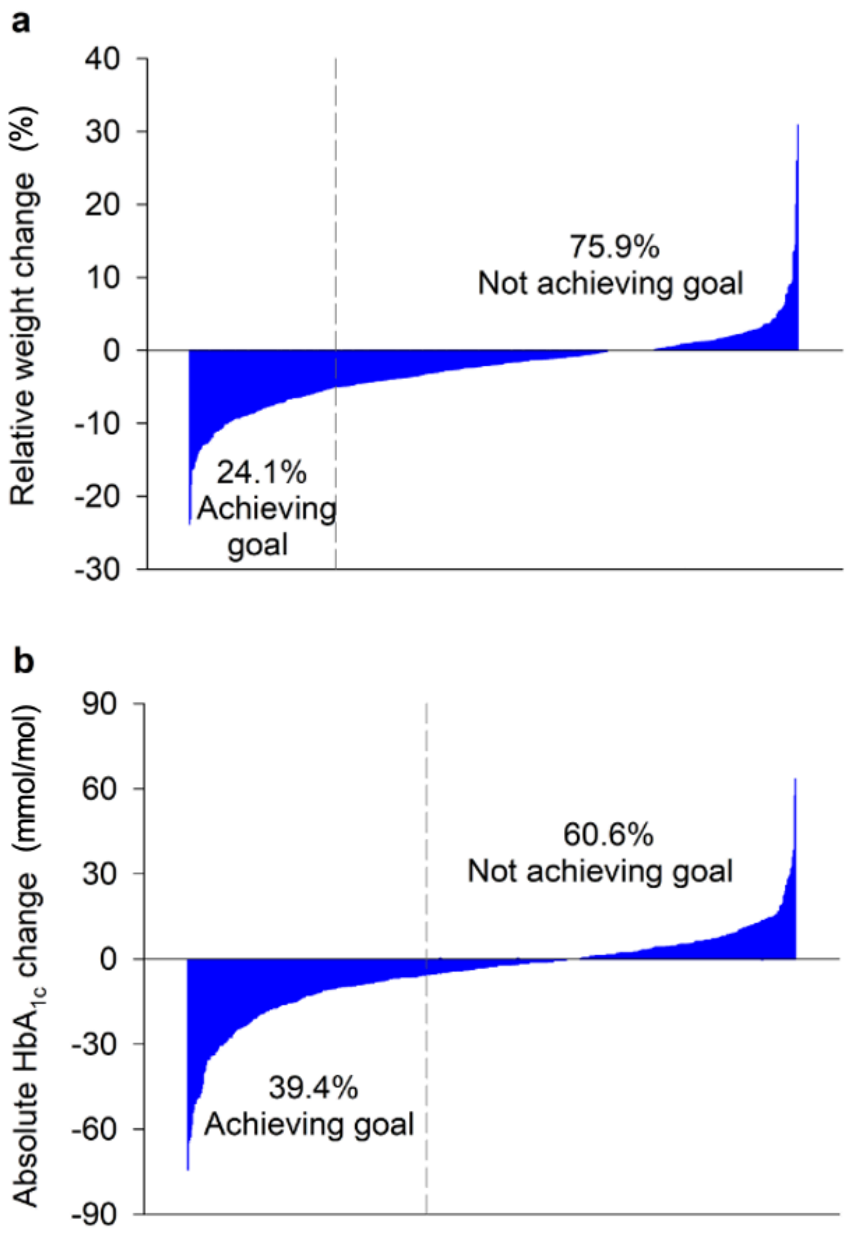

ESM Fig. 4 Associations with weight (**a**) or HbA_{1c} (**b**) reductions in the first six months upon initiation of liraglutide, semaglutide, or dulaglutide. Presented are standardized β-coefficients with 95% CI for the tested associations with baseline parameters from linear regression models. Positive β-coefficients indicate direct associations, negative β-coefficients indicate indirect associations.

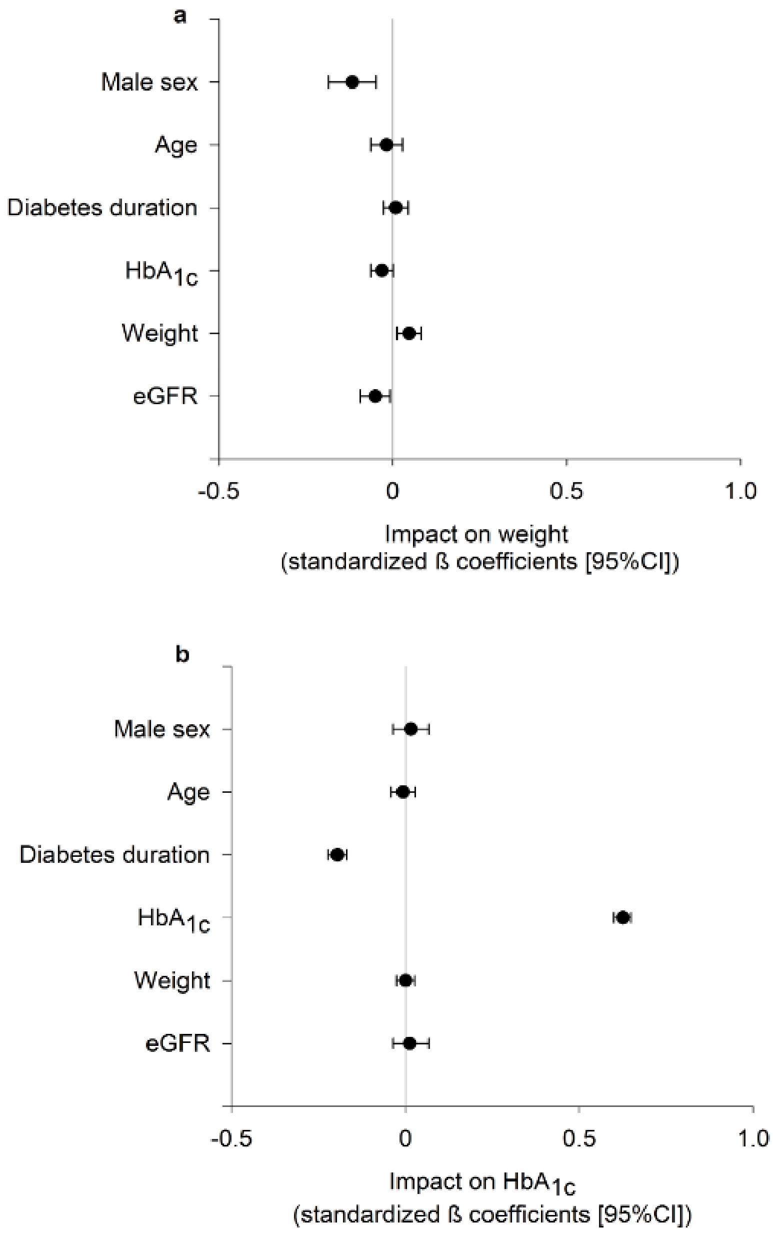

Supplement: Supplementary file 1 — ESM (PDF 524 KB) [file 125_2025_6448_MOESM1_ESM.pdf]
